# ATTITUDES AND BELIEFS ON INTERGENERATIONAL FAMILIAL CARE: IMPLICATIONS FOR FUTURE CARE POLICY IN LEBANON

**DOI:** 10.1093/geroni/igae098.1164

**Published:** 2024-12-31

**Authors:** Myriam Al Bcherraoui, Kristine Ajrouch, Toni Antonucci

**Affiliations:** University of Michigan, Ann Arbor, Michigan, United States; University of Michigan, Ann Arbor, Michigan, United States; University of Michigan, Ann Arbor, Michigan, United States

## Abstract

In a collectivistic society such as Lebanon, familism is a deeply rooted cultural value where family members learn to put the needs of the group over the self. Familial obligation is socially reinforced, and care is perceived as the principal responsibility of the family. In a rapidly aging society accompanied by economic hardships, older adults in Lebanon are living longer, often with health conditions, and family members assume the main responsibility of care. Solidarity in family relationships becomes increasingly important and may lead to many benefits in caregiving when they are positive, but they may also be equally harmful if conflictual. Understanding adults’ attitudes concerning differential care settings becomes an intricate state matter with ethical implications. Drawing on Bengtson’s Solidarity-Conflict Model of Generational Relationships, we assessed data from the Family Ties and Aging study (N=500) collected in Beirut, Lebanon from adults aged 18-91. The study examined 1) the extent to which age, gender, and education level influence beliefs held about elder care, and 2) whether parent-child relationship closeness predicts attitudes on state versus family care. Regression analyses indicated that greater relationship closeness to children predicts stronger beliefs favoring government-led care for personal and household matters. Older age and closeness to children also predicted parents’ disagreement in children’s obligational role in care, both personally and financially. This may relate to parents’ desire to decrease strain on family members and increase solidarity. Results will be discussed in light of the Solidarity-Conflict Model, emphasizing the importance of strengthening family ties through state interventions.

